# 2′-Fucosyllactose Effects on Preterm Growth, Tolerance, and Neurobehavior: A Double-Blind, Randomized, Placebo-Controlled Pilot

**DOI:** 10.3390/nu18152437

**Published:** 2026-07-26

**Authors:** Ethan A. Mezoff, Qing Duan, Nicholas J. Ollberding, Elizabeth J. Reverri, Rachael H. Buck, Bridget Barrett-Reis, Kimberly Yolton, Ardythe L. Morrow

**Affiliations:** 1 Division of Pediatric Gastroenterology, Hepatology, and Nutrition, Nationwide Children’s Hospital, The Ohio State University College of Medicine, Columbus, OH 43205, USA; 2 Division of Biostatistics and Epidemiology, Cincinnati Children’s Hospital Medical Center, Cincinnati, OH 45229, USA; qing.duan@cchmc.org (Q.D.); nicholas.ollberding@cchmc.org (N.J.O.); 3 Department of Pediatrics, Cincinnati Children’s Hospital Medical Center, University of Cincinnati College of Medicine, Cincinnati, OH 45229, USA; kimberly.yolton@cchmc.org; 4 Nutrition Science & Innovation, Abbott Nutrition, Columbus, OH 43219, USA; elizabeth.reverri@abbott.com (E.J.R.); bridget.reis@outlook.com (B.B.-R.); 5 Gut, Immunity, and Brain Health Platform, Abbott Nutrition, Columbus, OH 43219, USA; rachaelbuck64@gmail.com; 6 Department of Environmental and Public Health Sciences, University of Cincinnati College of Medicine, Cincinnati, OH 45267, USA; morrowa@ucmail.uc.edu

**Keywords:** 2′-Fucosyllactose, human milk oligosaccharides, preterm, neurobehavior, growth, tolerance

## Abstract

**Objectives**: Preterm infants confront multiple coexistent health risks and may not receive human milk (HM), which contains human milk oligosaccharides (HMOs), among other biologically active, beneficial components. 2′-Fucosyllactose (2′-FL) is the most abundant HMO in most HM. Interventional studies of formula with added HMOs have demonstrated multiple beneficial outcomes in healthy, term infants. **Methods**: This double-blind, randomized, placebo-controlled pilot study (NCT03306316) evaluated the effect of a 2′-FL supplement on growth, tolerance, and neurobehavior, as assessed by the Neonatal Intensive Care Unit (NICU) Network Neurobehavioral Scale (NNNS), in preterm infants. Eligible neonates born between 26 0/7 and 31 6/7 weeks gestational age (GA) were enrolled from three NICUs in the United States. Preterm infants consuming mother’s own milk or donor human milk were randomized to receive a supplement of 0.135 g/mL of 2′-FL or 0.05 g/mL dextrose (placebo) twice daily until 36 weeks corrected GA, NNNS assessment, or hospital discharge, whichever came first. **Results**: Of 42 participating preterm infants, 23 (55%) received 2′-FL and demonstrated no significant differences in growth in weight, length, or head circumference over 5 weeks. There were also no differences in stool frequency, adverse (AEs) or serious adverse events (SAEs), or neurobehavior (NNNS) between groups. **Conclusions**: This pilot study found that 2′-FL supplementation in preterm infants through 36 weeks corrected GA was safe and well-tolerated and supported adequate growth and neurobehavior.

## 1. Introduction

Infants born preterm confront multiple coexistent and interacting health risks, making premature birth costly, morbid, and potentially deadly. Adverse medical and neurobehavioral outcomes are inversely correlated to gestational age (GA) at birth and thus, degree of organ system immaturity. Human milk (HM) generally provides an optimal nutrition source for preterm infants when appropriately fortified to meet the unique needs of premature life. When mother’s own milk is unavailable or contraindicated, the American Academy of Pediatrics recommends use of pasteurized donor HM instead of formula [1,2,3]. Unfortunately, access to pasteurized donor HM is limited by availability and affordability. In situations where HM is inaccessible, preterm infant formula is needed to achieve and maintain enteral autonomy. To approximate the gap between HM and preterm infant formula and to improve health through use of HM fortifier, evaluation and use of differences in biologically active components are required. International guidelines on preterm nutritional care have listed several research priorities, including bioactive compounds such as human milk oligosaccharides (HMOs) [4].

HMOs comprise the third largest solid component of HM after lactose and lipids, respectively, and with concentrations exceeding 8 g/L from colostrum and human milk through the first month of life of preterm infants [5,6,7,8]. HMOs confer protection, resilience, and enhance organ development through multiple complementary mechanisms though specific concentrations and profiles vary by geography and lactation period, among other factors [9,10,11]. 2′-Fucosyllactose (2′-FL) is one of the most abundant HMOs secreted by women, consisting of an alpha1,2-linked fucose attached to lactose. One of the functions of HMOs including 2′-FL is as a prebiotic with clinical and preclinical evidence of multifarious benefit, now commercially available in many term infant formulas. Added 2′-FL to term infant formula is well-tolerated in clinical trials, supporting typical infant growth and gut health [12,13,14,15,16]. Preclinical evidence strongly suggests protection against NEC through various mechanisms including maintenance of mesenteric perfusion and inhibition of necroptosis [17,18,19]. Furthermore, it may convey resilience to such a severe disorder, reducing NEC-induced brain injury and strengthening the adaptive response to bowel resection [20,21]. Indeed, 2′-FL is generally an anti-inflammatory and immune-modulating HMO with clinical evidence of lowering inflammatory cytokine levels when supplemented in term infant formula or obtained directly from HM [22,23]. Preclinical evidence also supports a positive influence of 2′-FL on cognition [24,25,26].

We intentionally sought to conduct a prospective pilot study comparing 2′-FL supplementation to a placebo control among preterm infants born between 26 0/7 to 31 6/7 weeks gestation, stratified by HM source. The primary objective was to evaluate growth and tolerance of 2′-FL supplementation among preterm infants. Among infants randomized to receive the study supplement, 270 mg per day of 2′-FL was provided to give 2′-FL within the range of what preterm and term infants may receive [8,27,28]. Measures of growth, intake, output, and tolerance were monitored throughout the study. Neurobehavior was evaluated with the Neonatal Intensive Care Unit (NICU) Network Neurobehavioral Scale (NNNS) [29].

## 2. Materials and Methods

The study protocol, informed consent form, and recruitment materials were reviewed and approved by Nationwide Children’s Hospital Institutional Review Board (IRB) (Columbus, OH, USA; Tracking Number IRB17-00035). Before enrollment, parents or legally authorized representatives signed and dated the informed consent form and provided the Health Insurance Portability and Accountability Act or other applicable privacy regulation authorization.

The study was conducted in accordance with the protocol and consensus ethical principles, including the Declaration of Helsinki of 1975 and 2013 and applicable International Council for Harmonization Good Clinical Practice guidelines. This study was registered with ClinicalTrials.gov (ID NCT03306316) on 28 September 2017. Reporting of the study followed the Consolidated Standards of Reporting Trials (CONSORT) 2025 statement checklist for pilot and feasibility trials [30].

### 2.1. Study Design

This double-blind, randomized, placebo-controlled pilot study was conducted at three NICUs in Columbus, Ohio from July 2018 through May 2023. All infants admitted to a participating NICU after premature birth (26 0/7 to 31 6/7 weeks gestation) and of birthweight appropriate for GA (10th to 90th percentile on Fenton growth charts) were screened for eligibility and enrolled by day ten 10 life (baseline). At the time of enrollment, infants were included if mothers planned to provide mother’s own milk or consented to use of donor HM. Infants were not eligible for enrollment if they had a diagnosis of congenital anomalies, specific disease processes (i.e., NEC, neonatal sepsis, respiratory failure requiring mechanical ventilation, intraventricular hemorrhage), more than 2 days of antimicrobial use, postnatal corticosteroid use, maternal or infant treatment consistent with HIV therapy, plan for exclusive formula feeding, or maternal incapacitation evidenced by a positive drugs of abuse test during or immediately following pregnancy.

### 2.2. Study Interventions

After consent was obtained, infants were stratified by milk source (mother’s own milk or donor HM) and birthweight (above or below 1000 g) and then underwent electronic block randomization to study or placebo supplementation. The randomization schema was developed by the statistician before enrollment began; enrolling and assigning study staff did not have access to the completed allocation sequence. A block size of two was used within each stratum to ensure balanced allocation to treatment condition overall and within key subgroups. Investigators, study staff, hospital staff, and participants were blinded until the described analysis was complete. Administration of study or placebo supplementation began during the first 7–11 days of life and finished after completion of neurobehavioral testing, achievement of 36 weeks’ gestation, or at hospital discharge, whichever came first. Blinded two-fluid-ounce (fl oz) bottles containing the study or placebo supplements were received from Abbott Nutrition, stored, and dispensed daily by the Nationwide Children’s Hospital (NCH) Investigational Drug Studies pharmacy. One milliliter of study or placebo supplement was administered twice daily by mouth or via enteric tube. The study supplement dosage was 0.135 g/mL of 2′-FL dissolved in sterile water. This dosage was chosen to increase HMO intake to concentrations closer to term infants, but low enough to maintain oligosaccharide levels within a normal physiological range whether infants were receiving mother’s own milk, donor HM, or formulas without HMOs [8,27,28]. The study product was clear when in solution and had a pH of 6.2. The placebo supplement dosage was 0.05 g/mL dextrose dissolved in sterile water. The placebo product was clear when in solution and had a pH of 5.9. The study and placebo supplements possessed similar osmolality and visual appearance. They were provided until 36 weeks corrected GA, NNNS assessment, or hospital discharge, whichever came first.

### 2.3. Outcomes

The primary outcome was infant growth, as measured by anthropometric *Z*-scores between groups. Baseline anthropometric measures were recorded at enrollment. Weight was measured daily and length and head circumference was measured weekly throughout the study and plotted on the modified Fenton growth charts to determine the relationship to expected growth patterns [31].

A standardized feeding protocol was utilized by study NICUs for preterm infants ≤ 1500 g. In this protocol, feeding is initiated between day of life 1–3, starting at 10–20 mL/kg/day. Feeding advancements are made at 20 mL/kg/day. Around day of life 7, HMF is added to 22 kcals/fl oz then 24 kcals/fl oz the following day. Following this protocol, infants are expected to reach 135 mL/kg/day of enteral feeding by day of life 14. Tolerance measures included stool frequency and presence of adverse events during the study period (e.g., vomiting, apnea, bradycardia, and desaturation).

As an exploratory outcome, NNNS is a standardized neurobehavioral testing method that assesses classical neurological items reflecting central nervous system integrity, behavioral items, and signs of stress typical of preterm infants [29,32]. Infants were eligible for NNNS administration between 34 weeks and 0 days to 35 weeks and 2 days corrected GA among infants not requiring positive pressure respiratory support, ongoing isolette use for thermal regulation, or prompt unit discharge at study conclusion. Three examiners were trained and certified on proper administration and scoring of the NNNS. The 30 min exam includes assessment of muscle tone and movement, reflexes, attention and engagement, and behavioral and stress responses during testing, resulting in 13 summary scales that describe neurologic integrity and behavior. Stool samples were also collected and results will be published separately.

A Data Safety Monitoring Board (DSMB) was electively convened for this study and met routinely. Adverse events (AEs) were categorized and recorded, and serious AEs, monitored for 14 days after hospital discharge, were promptly reported to the IRB and DSMB.

### 2.4. Statistical Analyses

A sample size of *n* = 90 (*n* = 45 per group) was planned for this pilot study to detect potential differences in the primary outcomes of growth as measured by the *Z*-scores for weight, length, and head circumference at 35 weeks corrected GA. Calculations were based on achieving 80% power (1 − β) to detect a standardized mean *Z*-score difference as small as of 0.6 between groups to 35 weeks corrected GA and a standard deviation of 1.0 within a group for an independent samples *t*-test at α = 0.05 (two-sided).

Means with standard deviations (SDs) for continuous variables and frequencies with percentages for categorical variables were used to describe participant demographic and clinical characteristics by treatment group. Pearson’s chi-squared tests and Fisher’s exact tests were used to test for differences in the presence of AEs between the study and placebo groups. Generalized least squares regression was used to model changes over time in anthropometric measures according to treatment group. Models included terms for time, treatment, the time-by-treatment interaction, birthweight in grams, provision of any mother’s own milk vs. donor HM, and site and a continuous-time first-order autoregressive correlation structure for the residuals dependent on participant and time. Covariates were selected a priori to capture key prognostic factors and design considerations. Models were fit via restricted maximum likelihood and the coefficient and *p*-value from the *t*-test for the time-by-treatment interaction reflecting the estimated difference in the change in the *Z*-score per week for those randomized to 2′-FL when compared to placebo was of primary interest. Analyses were conducted in accordance with a modified intention-to-treat (ITT) analysis where all participants who were randomized and received at least one dose of 2′-FL or placebo were analyzed according to their group assignment, irrespective of adherence to the study protocol.

All analyses were performed using the R software environment for statistical computing and graphics (version 4.4.0). Generalized least squares models were fit using the Gls function in the rms package (version 6.8-2) and nlme package (3.1-165).

## 3. Results

Study procedures occurred from July 2018 to May 2023. Enrollment was paused from March 2020 to June 2020 in relation to coronavirus disease (COVID-19) restrictions and from October 2020 to October 2021 in relation to supplementation manufacturing. Of the 44 participants enrolled, 42 received the study or placebo supplementation and two were removed for ineligibility immediately after enrollment. Fourteen (33%) were female. The average GA, weight, length, and head circumference at birth were 30 weeks, 1379 g, 39.4 cm, and 27.5 cm, respectively (Table 1). Twenty-three participants (55%) received study supplementation and 19 (45%) received placebo supplementation. This pilot study was terminated before reaching enrollment goals due to prolonged enrollment time to reach reported numbers and in recognition of the pilot nature of the study.

### 3.1. Nutrition Source

All participants began the study receiving HM. None were routinely supplemented with probiotics. Feeding source was dynamic after enrollment despite intended human milk use, with infants transitioning among mother’s milk, donor milk, and formula throughout the study. When primary enteral nutrition source was summarized by study day, mother’s milk exposure predominated in both arms (placebo 426/496 days [85.9%]; 2′-FL 469/615 days [76.3%]), while donor milk without mother’s milk (placebo 19/496 days [3.8%]; 2′-FL 58/615 days [9.4%]) and exclusive formula (placebo 51/496 days [10.3%]; 2′-FL 88/615 days [14.3%]) comprised the remainder; no consistent temporal pattern of transitions subjects was apparent. Three required parenteral nutrition (PN) during the study. Two participants who had been randomized to the placebo group weaned from PN on study days 2 and 6. The third, who had been randomized to the study group, required 16 days of parenteral nutrition support during NPO status described below. There were no meaningful changes to enteral nutrition protocols utilized by enrollment sites during the study. The mean (SD) duration of study supplementation was 26.7 (12.4) days in the 2′-FL group and 26.1 (12.1) days in the placebo group (*p* = 0.87).

### 3.2. Anthropometrics

**Figure 1:**
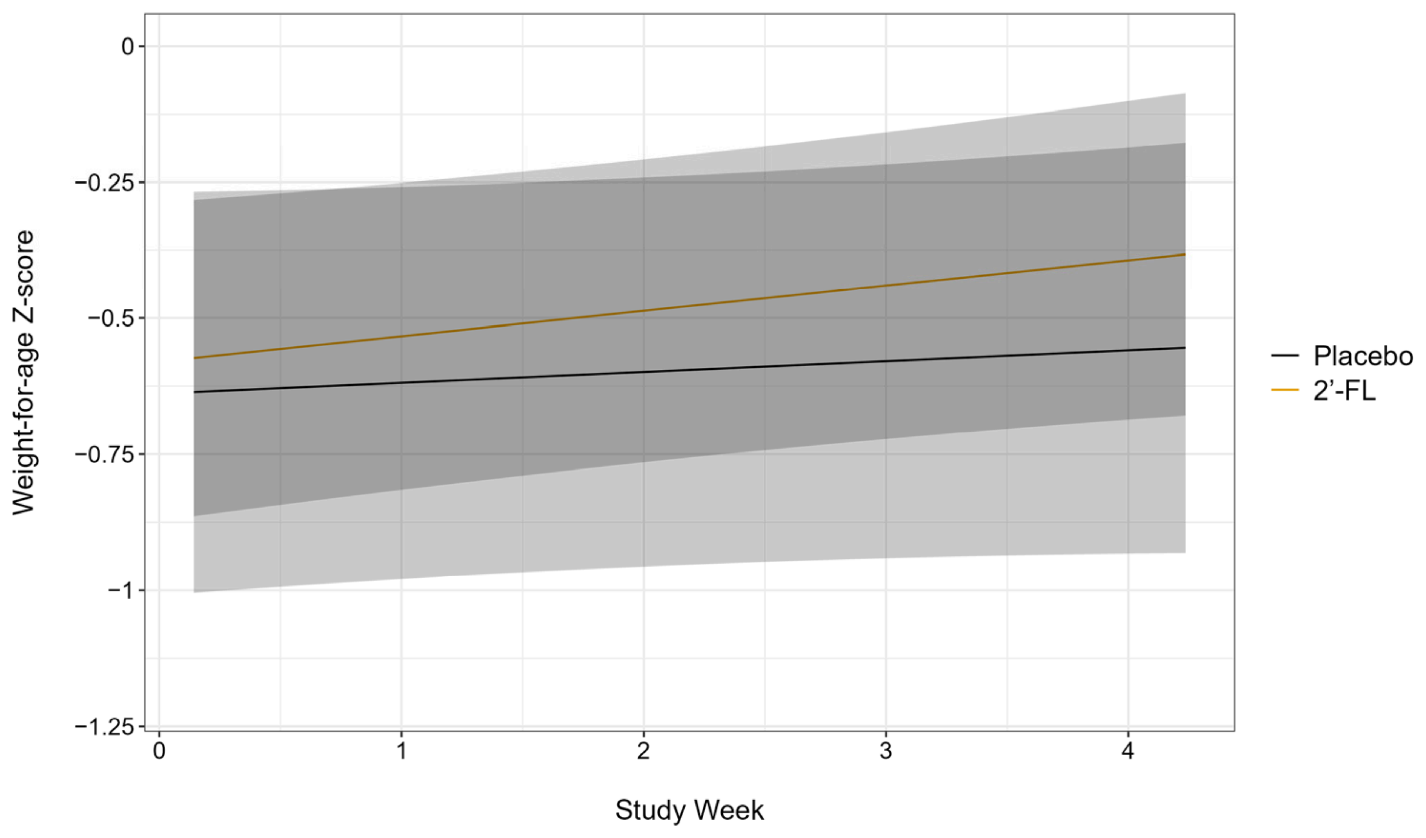
2′-Fucosyllactose effect on weight *Z*-scores of preterm infants over 5 weeks. Solid lines show the predicted mean weight-for-age *Z*-score according to study week and treatment arm. Shaded bands represent the 95% confidence interval for the predicted mean trajectories.

**Figure 2:**
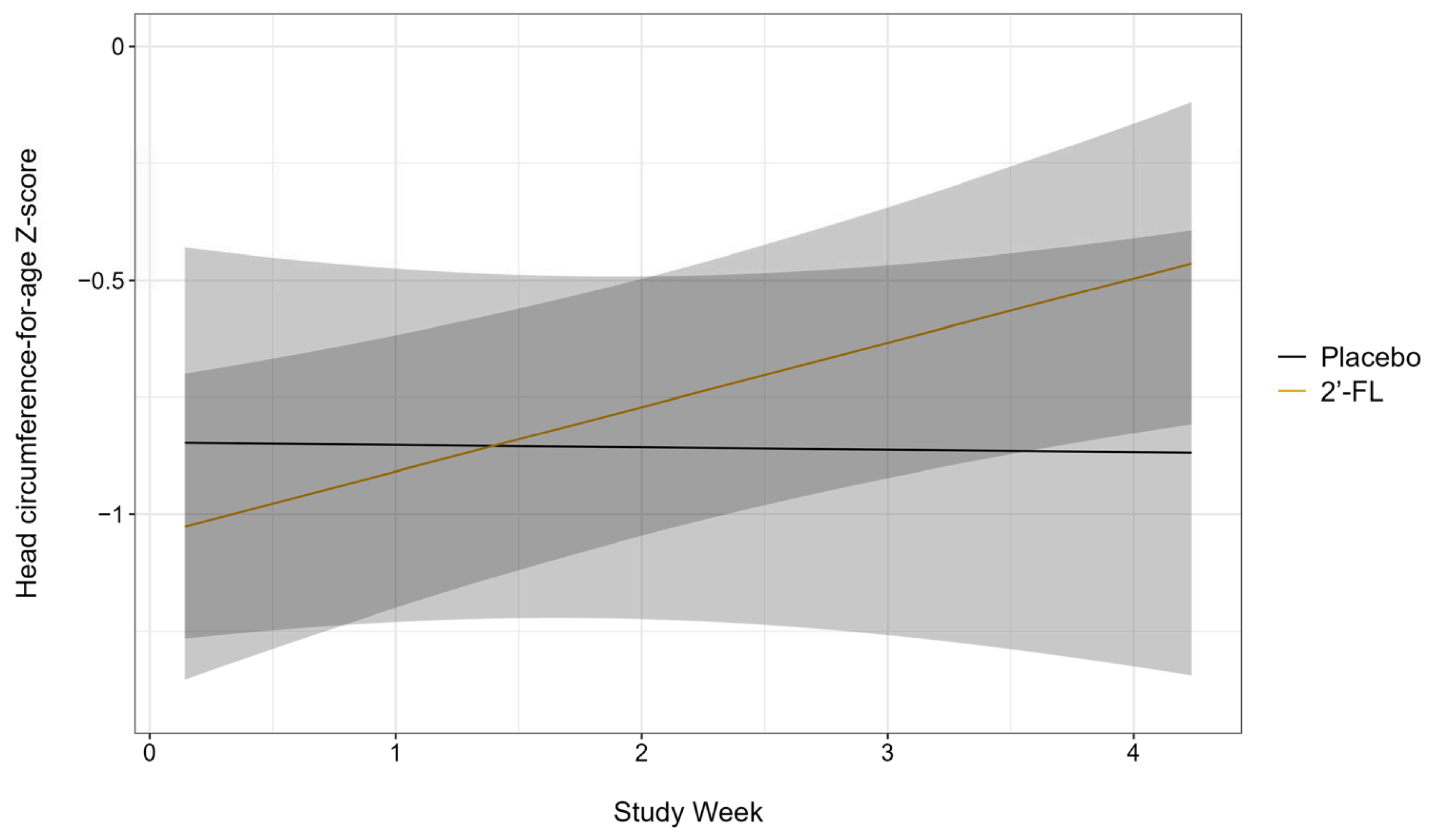
2′-Fucosyllactose effect on head circumference *Z*-scores of preterm infants over 5 weeks. Solid lines show the predicted mean head-circumference-for-age *Z*-score according to study week and treatment arm. Shaded bands represent the 95% confidence interval for the predicted mean trajectories.

There was an increase in the weight-for-age *Z*-score over time in both groups, with a non-significant trend toward higher *Z*-score in the study group (Figure 1, β = 0.03, SE = 0.04, *p* = 0.45 for the week-by-treatment interaction for 2′-FL vs. placebo). Normalized length-for-age declined over time in both groups (Figure S1, β = 0.01, SE = 0.07, *p* = 0.98). Head-circumference-for-age remained stable in the placebo group but trended toward increased normalized size in the study group (Figure 2, β = 0.14, SE = 0.08, *p* = 0.07).

### 3.3. Tolerance

Stool frequency declined by study week and did not differ by study group (*p* = 0.2753). Of 273 mild or moderate AEs that occurred during the study among all 42 participants, there were no statistically significant differences in the presence (Table 2), frequency, or intensity of events by group. There were four SAEs reported during the study, three of which occurred in the placebo supplement group. The first of these related to readmission with a viral upper respiratory illness 2 weeks after NICU discharge and 3 weeks after discontinuation of placebo supplementation. The second involved unilateral hearing screen failure in a participant with prior gentamicin exposure. The third involved hospital readmission for prematurity-related hypothermia occurring 6 days after NICU discharge and 10 days after discontinuation of placebo supplementation.

The fourth and only SAE to occur in a participant receiving study supplementation, was a collection of signs and symptoms leading to a suspicion for medical NEC. The participant was born at 29 weeks and 3 days gestation with birthweight of 1205 g and began receiving the study supplement 11 days after birth. At study entry, the participant was receiving full enteral nutrition with mother’s own milk fortified to 24 kcal/fl oz with HM fortifier. Three days after study entry, an episode of emesis and hematochezia prompted abdominal radiography which revealed, “gaseous distension of an elongated unfolded bowel loop in the RUQ [right upper quadrant]. No free air or convincing pneumatosis is identified.” The subject was transitioned to the NPO status with discontinuation of study supplementation. Empiric, broad-spectrum antimicrobial coverage was initiated. Subsequent radiographs remained free of pneumatosis, portal venous air, and free air. The infant remained euthermic with normal and stable vitals. Thus, medical NEC was suspected but not confirmed, and the infant had no lasting sequelae of this suspicion [33]. Of these four SAEs, none were judged to be probably or definitely related to the study or placebo supplementation at the time of event reporting.

### 3.4. Neurobehavior

Of 42 participants receiving study or placebo supplementation, 24 (57%) were eligible for neurobehavioral testing as an exploratory outcome. Among those who did not qualify, ineligibility was due to positive pressure respiratory support or ongoing isolette use for thermal regulation (*n* = 15) or prompt unit discharge at study conclusion (*n* = 3). Comparing groups who received and did not receive NNNS testing, the former trended toward a lower GA at birth (*p* = 0.079) with no statistically significant differences in birth *Z*-scores for weight, length, and head circumference between the groups tested and not tested (Table S1). There were significant differences in absolute anthropometric measurements indicating smaller size at birth in the infants not tested.

Among the 24 subjects that received neurobehavioral testing, 16 (67%) were in the 2′-FL group. There were no differences between groups regarding enrollment hospital, sex, GA at birth, age at the time of neurobehavioral testing, and absolute and normalized anthropometric measurement. There was also no difference in presence of AEs between groups. Scores on the NNNS were within the general ranges published for other cohorts of healthy newborn or preterm infants [34,35]. There were no clear differences in NNNS scores between groups for the 13 summary scales. While there was some indication that those in the 2′-FL group had slightly lower attention and self-regulation scores and slightly higher abnormal reflex scores, the small sample size and disproportion of study versus control infants precludes meaningful interpretation (Table 3).

## 4. Discussion

This double-blind, randomized, placebo-controlled pilot study demonstrated safety, growth, and tolerance of 2′-FL supplementation among infants born preterm. There were no adverse associations between supplemental 2′-FL, a prebiotic HMO that is also naturally present in HM of most lactating mothers, and the study outcomes. Growth was similar between the study and placebo groups among preterm infants receiving supplemental 2′-FL.

Tolerance of study and placebo products was similar. A single, serious AE occurred in the study group when abdominal distension and hematochezia prompted concern for medical NEC in an 14-day-old former 29-week infant. The risk of NEC is inversely related to GA and HM is known to be protective [36,37,38,39]. Among infants born moderately preterm, the risk of NEC is approximately 5–7% [40,41]. In this infant, the subsequent clinical course, including repeat imaging, did not ultimately support a diagnosis of NEC [33].

Among those undergoing neurobehavioral testing, there were no material differences between groups; however, the number of participants completing testing was limited and prevented appropriate statistical examination and generalizability. A growing number of observational studies have found select HMOs, including but not limited to 2′-FL, are positively associated with cognitive, language, and motor skill domains. Two observational studies have demonstrated mixed results of certain HMOs being associated with neurodevelopment (using Ages & Stages and Bayley-III, which were different measures than this pilot study) in preterm infants out to two years’ corrected age [42,43,44]. Rozé et al. found that both total and individual HMO concentrations in breastmilk fed to preterm infants between 1 and 7 weeks of age were not associated with later Ages and Stages scores [43]. Wejryd et al. found select HMO at 2 weeks of age, 3-FL and LSTb, were positively associated with Bayley Scales of Infant and Toddler Development [44]. Further, a review by Berger et al. summarized observational studies, finding that fucosylated and sialylated HMO are positively associated with neurodevelopmental outcomes at up to 24 months of age in those exposed as term infants [42]. The effect of Sialylated HMO on preterm neurodevelopment have not yet been published, to the author’s knowledge.

To our knowledge, only one other study of an HMO supplement (0.374 g/kg body weight/day of 2′-FL and Lacto-*N*-neotetraose (LNnT) provided over three doses/day) has been evaluated in 86 preterm infants [45]. That study had no significant differences between a supplement with 2 HMOs and placebo for weight-for-age *Z*-scores, days to reach full enteral feeds, stool consistency, and number of infections. The HMO-supplemented group had greater length- and head-circumference-for-age *Z*-scores at full enteral feeds days on 14 and 21 and at discharge, respectively, and decreased stool frequency after 1 week of full enteral feeds. This study of a supplement with two HMOs was similarly designed and found similar results to this pilot study. Collectively, these two studies confirm safety, growth, and tolerance in two different HMO supplements, both of which included 2′-FL, in preterm infants during the neonatal period. These results warrant further investigation in larger and longer-term clinical studies.

Future studies could be powered to evaluate for smaller differences, follow preterm infants for a longer period of time or follow those who are exclusively formula-fed, and to assess additional outcomes such as neurobehavioral outcomes at 18–24 months of age using developmental or behavioral testing appropriate for such an age. Future studies could also measure HMO concentrations directly and serially over time. These studies could include infants born in a setting of maternal incapacitation or disease populations of interest, such as preterm infants who have undergone substantial bowel resection. Strengths of this study were the strong study design of a randomized, blinded controlled trial and the biological relevance of the HMO concentration. For the first time, this pilot study evaluated 2′-FL supplementation alone in preterm infants and assessed neurobehavior in a subset of human milk-fed preterm infants supplemented with an added HMO.

This study has several relevant limitations. The first is the focus of this pilot study on otherwise healthy infants who began study product supplementation when enteral nutrition fortification began, whereas health benefits may be most pronounced when supplementation begins at feeding initiation or among infants with comorbid disease. The enrollment goal for this pilot study was not reached in a reasonable timeframe, in relation to two interruptions described above. Exclusion related to maternal incapacitation occurred at a much higher frequency than planned. Additionally, a prolonged pause in enrollment related to the COVID-19 pandemic and study supplement availability occurred. Together this resulted in a limited sample size of only 42 participants available for analysis and resultant reduction in statistical power and precision to detect differences between groups. Finally, competing demands of a structured randomized, controlled trial and the clinical needs of a population of vulnerable infants presented an unavoidable limitation. Three infants eligible for the NNNS assessment completed the study but were not administered the NNNS due to hospital discharge or return transfer to birthing hospital for ongoing, lower acuity care. Many others were ineligible related to ongoing need for continuous positive airway pressure.

## 5. Conclusions

In conclusion, 2′-FL is a prebiotic HMO found in the milk of mothers who express FUT2 and now also added in many term infant formulas. For the first time, this pilot study evaluated 2′-FL alone as a supplement in human milk-fed preterm infants and assessed neurobehavior in preterm infants fed an added HMO. Use in a preterm infant population was safe and well-tolerated in this pilot study and associated with adequate growth and neurodevelopment. Though larger studies are necessary to evaluate benefit and risk in this vulnerable population, this pilot study suggests that further research on HMOs is needed for this vulnerable population.

## Figures and Tables

**Table 1:** Participant characteristics at study entry.

Characteristic ^1^	Overall	Placebo	2′-FL
	N = 42	N = 19	N = 23
Hospital			
NCH	9 (21%)	6 (32%)	3 (13%)
OSU	15 (36%)	9 (47%)	6 (26%)
RMH	18 (43%)	4 (21%)	14 (61%)
Sex			
Male	14 (33%)	6 (32%)	8 (35%)
Female	28 (67%)	13 (68%)	15 (65%)
Gestational age	30.24 (1.30)	30.29 (1.20)	30.20 (1.40)
Birthweight			
Grams	1379 (255)	1372 (281)	1385 (238)
*Z*-score for age	−0.12 (0.63)	−0.18 (0.67)	−0.06 (0.60)
Birth length *			
Centimeters	39.44 (2.74)	39.36 (2.97)	39.51 (2.59)
*Z*-score for age	−0.21 (1.49)	−0.06 (1.47)	−0.34 (1.52)
Birth head circumference			
Centimeters	27.54 (1.73)	27.28 (1.89)	27.74 (1.60)
*Z*-score for age	−0.42 (1.57)	−0.63 (1.62)	−0.24 (1.55)

^1^ N (%); mean (SD). * out of 41 subjects measured. Abbreviations: NCH, Nationwide Children’s Hospital; OSU, The Ohio State University Wexner Medical Center; RMH, Riverside Methodist Hospital.

**Table 2:** 2′-Fucosyllactose effect on the presence of ≥1 mild or moderate adverse event among preterm infants.

Adverse Event During Study ^1^	Overall	Placebo	2′-FL	*p*-Value
	N = 42	N = 19	N = 23	
Regurgitation/Vomiting				0.826
Occurrence	14 (33%)	6 (32%)	8 (35%)	
Absence	28 (67%)	13 (68%)	15 (65%)	
Desaturation				0.661
Occurrence	28 (67%)	12 (63%)	16 (70%)	
Absence	14 (33%)	7 (37%)	7 (30%)	
Bradycardia				0.286
Occurrence	25 (60%)	13 (68%)	12 (52%)	
Absence	17 (40%)	6 (32%)	11 (48%)	
Other				0.199
Occurrence	40 (95%)	17 (89%)	23 (100%)	
Absence	2 (5%)	2 (11%)	0 (0%)	
Fever (≥38.0 °C)				>0.999
Occurrence	36 (86%)	16 (84%)	20 (87%)	
Absence	6 (14%)	3 (16%)	3 (13%)	
Anemia				0.673
Occurrence	36 (86%)	17 (89%)	19 (83%)	
Absence	6 (14%)	2 (11%)	4 (17%)	
Apnea				0.936
Occurrence	29 (69%)	13 (68%)	16 (70%)	
Absence	13 (31%)	6 (32%)	7 (30%)	
Respiratory Support				>0.999
Occurrence	40 (95%)	18 (95%)	22 (96%)	
Absence	2(4.8%)	1 (5.3%)	1 (4.3%)	
Thrombocytosis				0.492
Occurrence	40 (95%)	19 (100%)	21 (91%)	
Absence	2 (4.8%)	0 (0%)	2 (8.7%)	

^1^ N (%) Pearson’s chi-squared test or Fisher’s exact test.

**Table 3:** Summary statistics of neurobehavioral assessment with the NNNS among preterm infants receiving 2′-Fucosyllactose or placebo.

NNNS Score ^1^	Overall	Placebo	2′-FL	Difference *	95% CI
	N = 24	N = 8	N = 16		
Habituation ^2^	7.00 (5.83, 8.50)	7.67 (6.67, 8.50)	6.50 (5.33, 8.50)	0.31	−0.75, 1.4
Attention ^3^	6.14 (5.00, 6.43)	6.14 (5.00, 6.43)	5.64 (4.8, 7.29)	0.12	−0.79, 1.0
Arousal	3.14 (2.93, 3.57)	3.21 (2.79, 3.64)	3.14 (3.00, 3.50)	−0.02	−0.87, 0.83
Self-regulation ^4^	6.32 (6.00, 6.85)	6.92 (6.57, 7.30)	6.14 (5.85, 6.62)	1.0	0.05, 1.9
Handling ^3^	0.25 (0.13, 0.38)	0.25 (0.13, 0.38)	0.31 (0.13, 0.38)	−0.08	−0.98, 0.83
Movement	5.17 (4.79, 5.45)	5.18 (5.08, 5.53)	5.00 (4.63, 5.42)	0.70	−0.17, 1.6
Excitability	0.00 (0.00, 1.00)	0.00 (0.00, 0.50)	1.00 (0.00, 1.00)	−0.70	−1.6, 0.17
Lethargy	5.00 (4.00, 6.00)	5.00 (4.50, 6.00)	5.00 (4.00, 6.00)	0.12	−0.73, 0.97
Abnormal reflexes	5.00 (4.00, 7.00)	4.50 (2.50, 6.00)	5.50 (4.00, 7.00)	−0.52	−1.4, 0.34
Asymmetric reflexes	0.00 (0.00, 1.00)	0.00 (0.00, 0.50)	0.00 (0.00, 1.00)	−0.12	−0.96, 0.73
Hypertonicity	0.00 (0.00, 0.00)	0.00 (0.00, 0.50)	0.00 (0.00, 0.00)	0.82	−0.06, 1.7
Hypotonicity	0.00 (0.00, 0.00)	0.00 (0.00, 0.50)	0.00 (0.00, 0.00)	0.46	−0.40, 1.3
Stress signs	0.12 (0.06, 0.15)	0.09 (0.06, 0.13)	0.13 (0.08, 0.17)	−0.05	−0.90, 0.80

^1^ Median (Q1,Q3). * Standardized mean difference. ^2^ Placebo (N = 5); 2′-FL (N = 11). ^3^ Placebo (N = 7); 2′-FL (N = 14). ^4^ Placebo (N = 7); 2′-FL (N = 15). Abbreviations: NNNS, NICU Network Neurobehavioral Scale.

## Data Availability

The original contributions presented in this study are included in the article/Supplementary Material. Further inquiries can be directed to the corresponding author.

## Supplementary Materials

The following supporting information can be downloaded at: https://www.mdpi.com/article/10.3390/nu18152437/s1, Figure S1. 2′-Fucosyllactose effect on length Z-scores of preterm infants over 30 days. Table S1. Characteristics of subjects undergoing NICU Network Neurobehavioral Scale (NNNS) assessment. Table S2. Consolidated Standards of Reporting Trials (CONSORT) Checklist [46].

## Abbreviations

The following abbreviations are used in this manuscript:

2′-FL	2′-Fucosyllactose
FUT2	α1-2-fucoslyltransferase
AE	Adverse events
BPD	Bronchopulmonary dysplasia
COVID-19	Coronavirus disease
DSMB	Data Safety Monitoring Board
fl oz	Fluid ounce
GA	Gestational age
HMO	Human milk oligosaccharide
IRB	Institutional Review Board
ITT	Intention-to-treat
LNnT	Lactose-*N*-neotetraose
NCH	Nationwide Children’s Hospital
NICU	Neonatal Intensive Care Unit
NNNS	NICU Network Neurobehavioral Scale
NEC	Necrotizing enterocolitis
PN	Parenteral nutrition
ROP	Retinopathy of prematurity
RMH	Riverside Methodist Hospital
SD	Standard deviations
OSU	The Ohio State University Wexner Medical Center
